# Measuring social, economic, policy, and health system determinants of maternal health and survival: An urgent global priority

**DOI:** 10.1371/journal.pone.0317095

**Published:** 2025-01-10

**Authors:** R. Rima Jolivet, Jewel Gausman, Ana Langer

**Affiliations:** Women and Health Initiative, Department of Global Health and Population, Harvard University T.H. Chan School of Public Health, Boston, Massachusetts, United States of America; PLOS: Public Library of Science, UNITED KINGDOM OF GREAT BRITAIN AND NORTHERN IRELAND

## Abstract

In 2015, the World Health Organization (WHO) released global targets and strategies for reducing maternal mortality in the Sustainable Development Goal (SDG) period developed through broad stakeholder consultations. The targets and strategies identified in the “Strategies toward Ending Preventable Maternal Mortality (EPMM)” report are grounded in a systemic and human rights approach to maternal health and aim to address the broad spectrum of key social, political, economic, and health system determinants of maternal health and survival, as exemplified by 11 Key Themes. These upstream determinants of maternal survival are not well represented in maternal health measurement efforts, which tend to focus on service delivery. Thus, work was undertaken to develop a core set of maternal health indicators that could drive progress toward achieving the recommendations laid out in the EPMM Strategies that identified a menu of 25 indicators and 7 standard stratifiers related to the legal and policy environment, accountability mechanisms, inequities in access and quality, and empowerment of women, girls, families, and communities. Measurement experts have called for more research to ensure that indicators for monitoring maternal health, including its upstream determinants, are valid, which is critical if such measures are to be effective for driving and tracking progress toward ending preventable maternal deaths. This paper describes nine research reports emanating from seven discrete research studies to validate ten indicators in India, Ghana and Argentina that are compiled in a PLOS Collection with the aim of illustrating the breadth of the validation work, extracting some unifying themes and common findings, and discussing the implications for policy and practice they suggest.

## Introduction

In 2015, the World Health Organization (WHO) released the direction-setting report, “Strategies toward Ending Preventable Maternal Mortality (EPMM)” (EPMM Strategies) which outlines global targets and strategies for reducing maternal mortality in the Sustainable Development Goal (SDG) period [1]. This guidance document was developed through broad stakeholder consultations led by members of a global coalition spearheaded by the WHO. The targets and strategies identified in the report are grounded in research and a human rights approach to maternal health and are aimed at addressing the broad spectrum of key social, political, economic, and health system determinants of maternal health and survival, as exemplified by its 11 Key Themes (**Table 1**). As countries progress along the trajectory of the obstetric transition, and maternal deaths shift from being largely attributable to direct obstetric toward more indirect causes, addressing upstream, contextual factors is crucial for ending preventable maternal mortality and improving maternal health.

**Table 1:** EPMM 11 key themes.

**Guiding Principles**	1. Empower women, girls, families and communities
	2. Integrate maternal and newborn health, protect and support the mother-baby dyad
	3. Prioritize country ownership, leadership, and supportive legal, regulatory and financial frameworks
	4. Apply a human-rights framework to ensure that high-quality reproductive, maternal, and newborn health care is available, accessible and acceptable to all who need it
**Cross-cutting Actions**	5. Improve metrics, measurement systems, and data quality
	6. Prioritize adequate resources and effective health care financing
**Five Strategic Objectives**	7. Address inequities in access to and quality of sexual, reproductive, maternal and newborn healthcare
	8. Ensure universal health coverage for comprehensive sexual, reproductive, maternal, and newborn healthcare
	9. Address all causes of maternal mortality, reproductive and maternal morbidities and related disabilities
	10. Strengthen health systems to respond to the needs and priorities of women and girls
	11. Ensure accountability in order to improve quality of care and equity

The SDGs turn a wide lens on the goals and targets of global development, with a broad systemic focus on structural determinants of social, biomedical, and planetary wellbeing. Reflecting this broader perspective, an increasingly robust evidence base within the maternal and newborn health (MNH) measurement field shows that upstream determinants—which include social, economic, political, and health system factors—are critical to ending preventable maternal deaths [2–4]). But because they are significantly shaped by elements both across and beyond the health sector such upstream determinants are not as well represented in maternal health measurement efforts, which tend to focus on service delivery [5]. The advent of the SDGs also propelled a global measurement agenda.

To achieve and track progress toward SDG 3.1 there were numerous calls to action for strengthening maternal health measures and measurement approaches. [6–8] exhorting the global MNH community to demonstrate leadership in advancing the measurement agenda and ensuring its effectiveness. A seminal commentary outlined five principles, which came to be known as the “Kirkland Principles”. Five guiding principles (focus, relevance, innovation, equity, and global leadership) were proposed to direct such efforts. In 2020, at the five-year mark of the SDGs, the Kirkland principles were updated to include country ownership as an over-arching sixth principle [9, 10]. In addition, WHO convened an expert advisory group, the Mother Newborn Information for Tracking Outcomes and Results (MoNITOR) to improve maternal newborn health measurement and support national monitoring efforts. The group addresses the need to identify valid MNH indicators at global, regional, national and sub-national levels to ensure their usefulness towards achieving the SDGs and supporting strategies such as the UN Secretary General’s Global Strategy for Women’s, Children’s and Adolescents’ Health, the Every Newborn Action Plan and the EPMM Strategies.

Work was undertaken to develop a core set of maternal health indicators that could drive progress toward achieving the recommendations laid out in the EPMM Strategies. From 2016–2017, to assist in reaching SDG target 3.1., one hundred-fifty experts from more than 78 organizations worldwide participated in an extensive, multi-step consultation using five rounds of modified Delphi process. They systematically appraised existing measures and identified a menu of 25 indicators and 7 standard stratifiers tailored to 11 Key Themes in the EPMM Strategies (**Table 2**) [4]. The selected indicators relate to the legal and policy environment, accountability mechanisms, inequities in access and quality, and empowerment of women, girls, families, and communities. Although the majority were already in use, like most policy and health system-level indicators, few had ever been systematically assessed for validity.

**Table 2:** Panel 1. Indicators & Stratifiers tailored to 11 Key Themes in the EPMM Strategies.

Presence of laws and regulations that guarantee women aged 15–49 access to sexual and reproductive health care, information, and education
Gender Parity Index (GPI)
Whether or not legal frameworks are in place to promote, enforce, and monitor equality and non-discrimination on the basis of sex
Presence of protocols/policies on combined care of mother and baby, immediate breastfeeding, and observations of care
Maternity protection in accordance with ILO Convention 183
International Code of Marketing of Breastmilk Substitutes
Costed implementation plan for maternal, newborn, and child health
Midwives are authorized to deliver basic emergency obstetric and newborn care
Legal status of abortion
Proportion of women aged 15–49 who make their own informed decisions regarding sexual relations, contraceptive use, and reproductive health care
Geographic distribution of facilities that provide basic and comprehensive emergency obstetric care (EmOC)
Presence of a national set of indicators with targets and annual report to inform annual health sector reviews and other planning cycles
Maternal death review coverage
Percentage of total health expenditure spent on reproductive, maternal, newborn, and child health
Out-of-pocket expenditure as a percentage of total expenditure on health
Annual reviews are conducted of health spending from all financial sources, including spending on RMNCH, as part of broader health sector reviews
Health worker density and distribution (per 1000 population)
Coverage of essential health services
If fees exist for health services in the public sector, are women of reproductive age (15–49) exempt from user fees for [MH-related health] services
Demand for family planning satisfied through modern methods of contraception
Availability of functional emergency obstetric care (EmOC) facilities
Density of midwives, by district (by births)
Percentage of facilities that demonstrate readiness to deliver specific services: family planning, antenatal care, basic emergency obstetric care, and newborn care
Civil registration coverage of cause of death (percentage)
Presence of a national policy/strategy to ensure engagement of civil society organization representatives in periodic review of national programs for maternal, newborn, child, and adolescent health (MNCAH)
Standard Equity Stratifiers
Wealth
Area of residence: urban/rural
Area of residence: geographic region
Level of education: women’s education level
Age
Transparency Stratifier Available in the public domain

In 2019–2020, an influential landscape analysis and a seminal definitional framework for indicator validity in the specific context of maternal newborn health measurement were published in work commissioned by the WHO MoNITOR group [11, 12]. These papers underscored the need for rigorous research methods and testing efforts to ensure that indicators for monitoring maternal health, including its upstream determinants, are valid–that is, that they capture a concept or phenomenon that is demonstrably meaningful to maternal health and do so accurately. Their validity is critical if such measures are to be effective for driving and tracking progress toward ending preventable maternal deaths.

**Fig 1:**
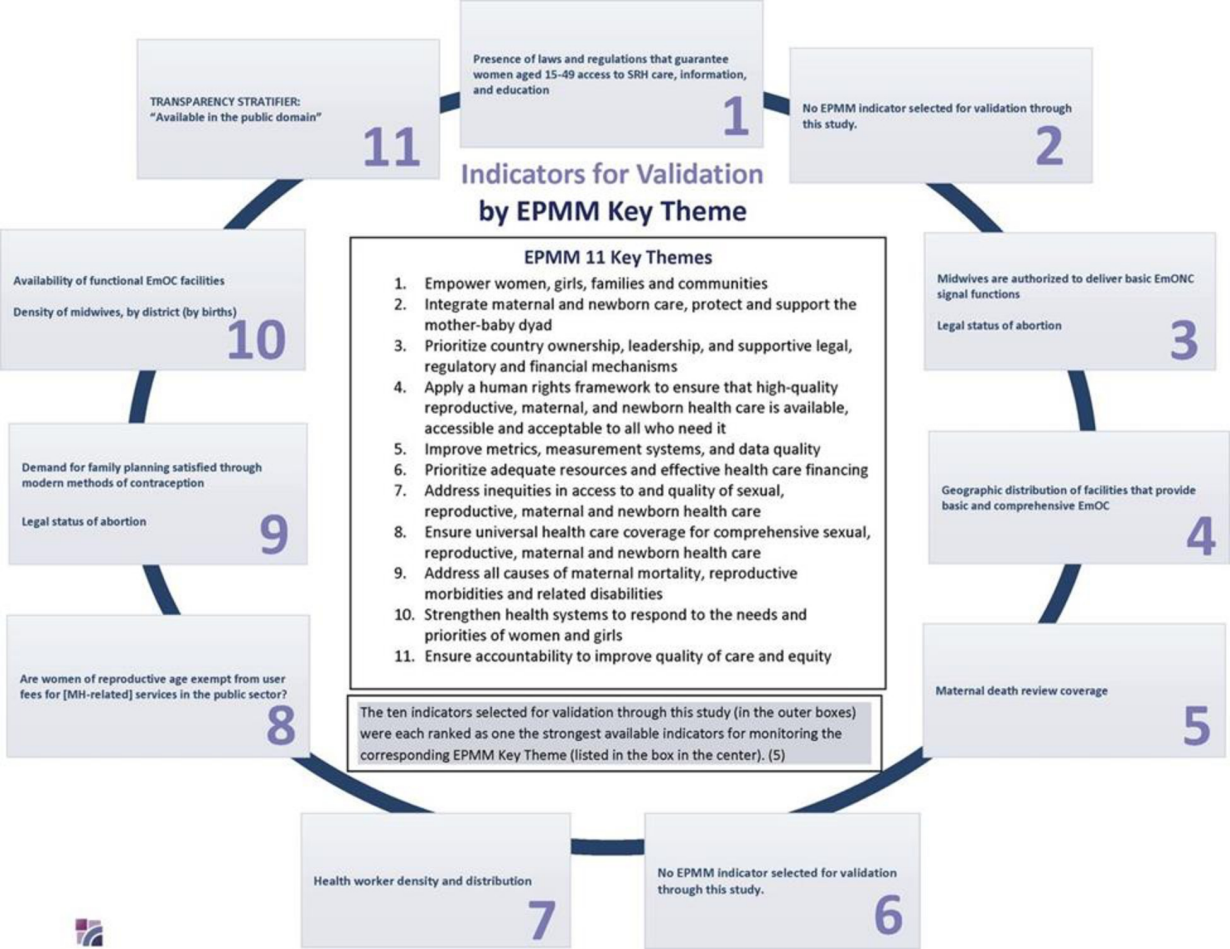
EPMM indicators validated by the IMHM project research studies.

With that goal, the Improving Maternal Health Measurement Capacity and Use (IMHM) Project, an international partnership led by the Women & Health Initiative at the Harvard T. H. Chan School of Public Health, conducted seven discrete research studies to validate ten EPMM indicators in India, Ghana and Argentina (**Fig 1**). The methodological reports and original research findings from the individual studies within the research project are compiled in a PLOS Collection with the goal of documenting the process and outcomes of this work. This paper provides an overview of all the studies in the collection with the aim of illustrating the breadth of the validation work, extracting some unifying themes and common findings, and discussing the implications for policy and practice they suggest. (**Table 3**).

**Table 3:** Panel 2. Seven Discrete Research Studies to Validate Ten EPMM Indicators.

	Indicator	Validation Question	Participant Data	Facility Data	Other Data Sources
1	Legal status of abortion	*How does the law*, *as expressed in the national statute*, *compare to the Countdown indicator metadata and to the information available on the WHO Global Abortion Policies Database for the country*?	-	-	National records (laws, regulations, policies)
		*Is there evidence that providers are consistently applying the law for each ground on which abortion is legal*?	Abortion Providers	-	-
2	If fees exist for health services in the public sector, are women of reproductive age (15–49) exempt from user fees for [MH-related health] services	*Does the free care law or policy in the country provide all of the categories of services included in the indicator free of charge or fees to users*?	-	-	National records (laws, regulations, policies)
		*For the categories of services that should be free according to the law/policy in the country*, *is there evidence that women are paying user fees for them*?	Women in Facilities (15–49 years)	-	-
			Companion of choice	-	-
			Chief Financial Officer	-	-
		*If evidence is found that demonstrates that women are paying for services that are supposed to be free according to the law/policy in the country*, *is there evidence that user fees are being levied in a systematically differential way to women*?	-	-	Stratification of study data
3	Density of midwives, by district (by births) vs Health worker density and distribution, disaggregated by midwives (per 1,000 population)	*How does the scope of practice of midwife/midwifery professional on record in country compare to ILO definition and ICM competencies description*?	-	-	National records on scope of practice (government sources)
		*What proportion of practicing midwives meet the ICM competencies description*?	Midwives/Midwifery Professionals	-	-
		*How does the value of the estimate differ based on the denominator used*?	-	-	Census and birth registry data
		*Does the national regulatory framework in country that authorizes midwives/MPs to deliver EmONC match what has been reported for this indicator for all 7 signal functions*?	-	-	National records, Professional association standards
		*For signal functions that midwives/MPs are authorized to perform according to national regulations*, *is there evidence they have performed these tasks in EmONC-designated birthing facilities*?	Midwives/Midwifery Professionals	-	-
4	Midwives are authorized to deliver basic emergency obstetric and newborn care	*Is there evidence from facilities designated as B/CEmONC to demonstrate that they have performed all 7/9 signal functions in last 3 months as defined in the metadata for these indicators*?	-	Facility assessments on signal functions	National Facility Assessment Reports
					-
		*How does the value of the indicator differ based on the denominator used*: *500*,*000 population/district vs*. *20*,*000 birth/district vs*. *travel time (<2 hours for BEmONC and <12 hours for CEmONC)*?		-	Census data; Birth registry; GIS data
5	Maternal death review coverage	How does evidence from facility-level on MDRs compare to the reported coverage of MDRs at district level?	-		-
				Facility-level MDR committee reports	District-level review coverage reports
		*How does the number of facility deaths captured through review of HMIS data compare to the number of deaths reported*?		Facility chart review / patient register	-
				-	HMIS Data
		*How does the value of the indicator reported compare to the value calculated using primary data*?		-	Analysis of study data
6	Demand for family planning satisfied through modern methods of contraception	*How does a direct measure of satisfaction of a woman’s demand through modern methods of contraception (women self-report) compare to the result provided by the DHS algorithm*?	Women in the community (15–49 years)	-	**-**
		*How does the value of the indicator vary based on new data source/estimation method compared to established source/method*?	-	-	Analysis of study data
7	Presence of laws and regulations that guarantee women aged 15–49 access to sexual and reproductive health care, information, and education	*To what extent do existing laws guarantee that the 13 different components are accessible*, *as reflected in the indicator*?	-	-	UNFPA Module II Inquiry Survey data, publicly available national data (laws, regulations, policies)

### Study summaries

The article by Williams et al., “Improving measures of access to legal abortion: A validation study triangulating multiple data sources to assess a global indicator” [13] looks at a core global indicator for addressing maternal mortality due to unsafe abortion. From 2010–2014, 56 million induced abortions were reported by the WHO; roughly 25% of all pregnancies are terminated, and 5–13% of all maternal deaths globally per year are attributable to unsafe abortion. Barriers to safe abortion include restrictive laws and unnecessary requirements that delay, stigmatize, or otherwise deter women from accessing abortion services. Further, the application of laws or requirements for accessing abortion services may vary by provider or facility creating additional barriers at the point of care. The aim of this study was to verify that the "legal status of abortion” indicator reported globally by each country accurately reflects the laws and statutes on record; and to look for variation at the provider- and facility-level of the application of the legal grounds, and thus the accessibility of induced abortion. This analysis revealed discrepancies between data reported by the global monitoring and accountability mechanisms and the domestic policy reviews, even though all referenced the same source documents. Further, provider surveys unearthed important context-specific barriers to legal abortion not captured by the indicator, including widespread conscientious objection and imposition of restrictions at the provider’s discretion. Taken together, these findings denote weaknesses in the validity of the indicator “legal status of abortion” as a proxy measure for access to safe abortion, as well as inaccuracies in data reported to global monitoring mechanisms. This information provides important groundwork for strengthening indicators for monitoring access to abortion and for renewed advocacy to assure abortion rights worldwide. By examining how the laws and requirements to access abortion services are applied at the facility- and provider-level, this study adds to global efforts focused on strengthening country-level transparency and accountability in ensuring access to safe abortion. by validating whether the laws and policies on record reflect the on-the-ground reality that women face when seeking abortion services.

The article by Odikro et al., “Validating implementation of an indicator reporting policies and laws on free public maternal health-related services in the era of universal health coverage: A multi-country cross-sectional study” [14] https://doi.org/10.1371/journal.pone.0299249 explores whether the policy indicator, “Are the following (enumerated, maternal health-related) health services provided free of charge at point of use in the public sector for women of reproductive age?” is a valid measure of universal health coverage (access to essential maternal healthcare free from financial harm), a primary strategic objective toward ending preventable maternal mortality. Pregnant women, adolescents, and children are particularly vulnerable to access constraints and related financial hardship as they more often lack the independent means to pay for health care services; thus, universal coverage policies prioritize free access to maternal-health related services. Further, despite the existence of free care policies and laws, formal or informal charges may accrue to women for services that are reportedly free, whether overtly due to health system deficiencies that force facilities or providers to charge for essential commodities, or covertly because of demands for informal payments. The aim of this study was to compare evidence from multiple data sources to verify that no charges, formal or informal, are assessed for the services included in the indicator that should be free by law, and to describe variance between these data sources. Women interviewed in Argentina, Ghana, and India. reported having to pay for all categories of services designated as free. The highest prevalence of out-of-pocket expenditures reported by service category in each setting was for cesarean section in Argentina (26%, 24/92); family planning in Ghana (78.4%, 69/88); and postnatal maternal care in India (94.4%, 85/90). A significant proportion of financial officers—9.1% (2/22) in Argentina, 64.1% (93/145) in Ghana, and 29.7% (47/158) in India—reported that women sometimes must pay for services that should be free in the facilities where they work, reflecting known health system deficits. Across the three countries, self-reports of out-of-pocket expenditures were significantly associated with district/province and educational status of women, while they were additionally associated with. wealth quintile in Argentina and age in India. Although free care laws were largely accurately reported in the global MNCAH policy database, the study found that women absorbed both direct and indirect costs and made both formal and informal payments for services designated as free. Thus, the policy indicator did not provide a valid reflection of UHC in the three settings. UHC is an important driver toward improving women’s health and survival in many LMICs. Ensuring consistency between laws and policies guaranteeing UHC and their application in practice are necessary to ensure the goals of equitable access and coverage are fulfilled.

Best evidence, as well as global guidance from entities like WHO and UNFPA, highlights the critical role of midwives in ensuring skilled birth attendance and quality maternal newborn care to ensure optimal maternal health and improve survival. An adequate midwifery workforce is critical for ending preventable maternal/newborn mortality. Three articles in the series assess the validity of indicators useful for monitoring effective coverage of midwifery practice and midwifery professionals.

The article by Ramesh et al., “Authorization of midwives to perform basic emergency obstetric and newborn care signal functions in Argentina, Ghana, and India: A multi-country validation study of a key global maternal newborn health indicator” [15] https://doi.org/10.1371/journal.pone.0283029 sought to validate this core policy indicator tracked in global monitoring frameworks. Whether a national policy allows midwives to deliver the seven functions of basic emergency obstetric and newborn care is used to track availability of essential maternal and newborn health interventions, yet little evidence supports whether such data are captured accurately, or whether midwives’ authorization demonstrates convergence with their skills and actual provision of basic emergency services. The authors detected discrepancies between data reported in the global monitoring frameworks and the national policy and regulatory frameworks in all three countries. In none of the countries were midwives authorized to perform all seven BEmONC signal functions, the reference standard for this indicator. Wide variations were found in midwives’ authorization to perform signal functions as documented in national policies, laws, and regulations and their self-reported skills and actual performance within the past 90 days. The percentage of midwives who reported that, in the past 90 days, they had performed all signal functions for which they were authorized per country-specific regulations was 17% in Argentina, 23% in Ghana, and 31% in India. Additionally, midwives in all three countries reported performing some signal functions that the national policy and regulatory frameworks did not authorize them to perform. These findings suggest limitations in criterion and construct validity for the indicator in Argentina, Ghana, and India. Discrepancies between global monitoring frameworks and national regulatory frameworks represent threats to criterion validity. Further, authorization did not consistently translate into obtaining the required skills or actually providing basic emergency services, undermining the construct validity of this measure. Findings suggest the need to re-examine the emergency interventions that should be included as BEmONC signal functions as some signal functions, such as assisted vaginal delivery, may be obsolete based on current practice patterns.

Two further articles in the series assess the validity of indicators used for tracking whether a country’s health workforce includes a sufficient number of midwives to meet population needs. Two measures used for planning and monitoring midwifery workforce adequacy are: 1) “Health worker density and distribution (per 10,000 population)”; and 2) “Density of midwives by district (by births)”. The optimal formulation of an indicator to measure this construct is unknown. Measurement of these indicators is complicated by differing definitions of a midwife, training and competencies, and authorization to perform tasks. Furthermore, the estimates of density and distribution of midwifery personnel use different parameters for both numerator and denominator, and neither considers a midwife’s competencies or authorization to deliver essential services.

The article by Chakraborty et al., “Validating midwifery professionals’ scope of practice and competency: a multi-country study comparing national data to international competencies” [16] https://doi.org/10.1371/journal.pone.0286310 focused on validating the numerator of indicators seeking to estimate midwifery density. It aimed to compare the regulated scope of practice for midwifery professionals in each country to international reference standards obtained from the International Labor Organization’s (ILO) international standard classification of occupations (ISCO-8) for midwifery professionals and midwifery associate professionals. It also assessed the degree to which midwifery professionals in each country reported having the skills and practicing the behaviors reflected in the International Confederation of Midwives (ICM) Essential Competencies for Basic Midwifery Practice. Such comparisons sought to assess the validity of the count of midwifery professionals used in indicators of midwifery density, since a reliable and valid count of midwives to meet population need assumes that each midwife counted is authorized to deliver the same interventions and has the capability to perform them competently. The study found that national scope of practice policies were limited compared to the ILO classification in Argentina and India, but matched the global reference standard in Ghana. Similarly, national standards for midwifery competency partially reflected the ICM skills across categories in Argentina (range 11% to 67%) and in India (range 74% to 100%) while the Ghana Health Service national source documents aligned completely with the ICM skills across categories. Findings from 1,266 midwifery professionals surveyed in the three study countries showed considerable variation in reported competency for skills and behaviors across three ICM categories: Category 2 (pre-pregnancy and antenatal care (ANC)), Category 3 (care during labor and birth), and Category 4 (ongoing care of women and newborns). Midwives predominantly reported having skills and behaviors that matched the sub-categories of ICM competencies around labor and childbirth; however, skills in the competencies for pre-pregnancy and ANC, and PNC and care of the newborn were reported by fewer midwives. Higher proportions of midwives reported receiving skills through in-service training and on job experience than through pre-service education. These findings suggest a considerable variation in national midwifery scopes of practice and competencies compared to the global reference standards. Estimating the density of midwives—the number needed to ensure effective coverage and quality of midwifery interventions—is predicated on a valid numerator. Only if midwifery professionals in the count can reliably exercise the same scope and skills with comparable competency can we assume that the number of midwives in the numerator is a valid indicator of an adequate midwifery workforce. The study results suggest gaps in skills and behaviors essential for midwifery practice across the full continuum of maternity care. However, they also suggest that the potential for expanded authorization and improved education and training to meet reference standards has not been fully realized. The authors note that the complex, composite descriptions of skills and behaviors included in each sub-component for each category of the ICM competencies make them difficult to use as measures of midwifery competency with any precision. Although the universally recognized global standard, this study demonstrates that the ICM competencies are not defined or structured in such a way to serve as valid measures for assessing workforce competency. A simplified, content-validated measurement system is needed to facilitate evaluation of the competency of the midwifery workforce.

The article by Gausman et al., “Measuring adequacy of the midwifery workforce: Exploring the density and distribution of midwives in three low- and middle-income countries using cross-sectional and geospatial data” [17] sought to strengthen measurement of midwifery workforce adequacy. Three aspects of adequacy were reflected: density (number to meet need), distribution (accessibility), and both competency and authorization to provide essential care (availability). The authors compared the value of estimates derived from two indicators designed to measure the same construct (density and distribution of midwives and midwifery professionals), to explore whether the two measures are consistent and their values track reliably with each other (convergent validity), or whether adjusting the numerator and/or the denominator could provide a more valid estimate of this construct. To do so, they calculated the density and distribution of facility-based midwifery professionals iteratively, varying the numerator by using an estimate of midwifery professionals counted according to job description, compared to those reporting most, or all, ICM competencies essential for basic midwifery practice; and varying the denominator to calculate the value of midwives by 10,000 population by district, by births per district, and by pregnancies per district. Variance in the estimates obtained using each estimation method was reported. In using different values for the numerator, the resulting indicator values showed how factoring in competency impacts the measure of effective coverage of midwifery professionals, reducing the density of effective midwifery coverage from within range of available targets to well below, or in some cases to zero. For the denominator, the study presented the variance in the value of the estimate, in some cases of orders of magnitude, using an array of population parameters that are potentially more meaningful than total population. The optimal value for this indicator is unknown; however, the study results suggest low convergent validity between two indicators that aim to represent the same construct—the density and distribution of midwives sufficient to meet population needs, when those needs are estimated based on total population or births. Importantly, this study shows that the underlying parameters significantly affect the value of the estimate. To ground the indicator in empirical evidence of effective coverage, future research should compare various estimates of midwifery density to health system process and outcome measures. Furthermore, testing the optimal denominator for evaluating adequacy of the midwifery workforce is useful since they provide quality maternal and newborn care services outside the context of childbirth.

Another article by Gausman et al., “Validating indicators for monitoring availability and geographic distribution of emergency obstetric and neonatal care (EmoNC) facilities: A triangulation study of health system, facility, and geospatial data” [18] https://doi.org/10.1371/journal.pone.0287904 assessed the validity of indicators used for monitoring availability and geographic distribution of EmONC by exploring multiple dimensions of availability: availability of all B/CEmONC signal functions within designated B/CEmONC facilities, and whether there are sufficient B/CEmONC facilities to meet the needs of the population (coverage). Several important challenges to measuring the availability and distribution of EmONC services exist. Reports suggest that many more facilities are designated as B/CEmONC than consistently demonstrate the capacity to function as such; if true, this challenges the validity of measures of B/CEmONC availability. Furthermore, the indicators as currently defined examine the number of designated and functional facilities per 500,000 population, but other denominators may provide more meaningful measures of service availability. The study compared estimates that emphasize different dimensions of the availability of facilities providing emergency obstetric and newborn care and that are based on different measurement approaches and different data sources to explore their external consistency or convergent validity. Facility performance of BEmONC and CEmONC signal functions varied considerably across and within study settings. Performance of all B/CEmONC signal functions as well as 24/7 care, also varied considerably among facilities designated as B/CEmONC in each country. Performance of all seven basic signal functions and two additional comprehensive signal functions was also fairly limited among CEmONC-designated facilities in the study countries. Considering a facility’s staffing and availability of essential medicines, a larger percentage of CEmONC-designated facilities provided 24/7 care and had greater availability of essential medicines than facilities that were BEmONC-designated or did not have an EmONC designation. Across all countries, the vast majority of BEmONC-designated facilities were found to be neither fully nor partially functional according to our study definitions. In Ghana, only 4.8% of all BEmONC-designated facilities were fully functional, and three districts did not have either a fully or partially functional BEmONC facility. When calculating the indicator using EmONC-designated facilities in the numerator regardless of their functionality, a greater number of study areas reach the target of 5 BEmONC facilities per 20,000 births than for 500,000 total population. The magnitude in the difference between indicator estimates obtained based on total population versus births was uneven across study areas within and across countries. However, after considering either full or partial functionality of facilities at the BEmONC-level, the value of the indicator changed dramatically in all countries and sub-national study areas. At the CEmONC-level, there was less variation across sub-national study areas when changing the value of the numerator to incorporate facility functionality than at the BEmONC-level. In many districts, even if there is only one partially or fully functional EmONC facility, the bulk of the population is within two-hours travel time to the facility. The study highlights significant differences in the value of estimates of sufficient EmONC coverage derived from country data depending on the definition of the indicator and measurement approach used. The optimal definition and calculation of a core measure to capture this construct is subject to uncertainty and the global reference standard indicators are currently under revision. This study applied primary data to generate evidence that can help inform the debate. To provide a valid measure of effective coverage of EmONC, the construct for measurement via this indicator should extend beyond the narrowest definition of availability of emergency facilities to include evidence of regular performance of emergency signal functions, facility readiness to do so reliably and effectively, and appropriate geographic distribution for accessibility to functional facilities by the best representation of the population in need.

The article, “Validating the indicator “maternal death review coverage” to improve maternal mortality data: A retrospective review of district, facility, and individual medical record data” by Gausman et al. [19] https://doi.org/10.1371/journal.pone.0303028 explored threats to validity in both the numerator and the denominator in this indicator. In the denominator, both under-reporting and misclassification of maternal deaths present barriers to the accurate quantification of maternal mortality. Maternal death review data may be used to address these issues with death registration, but the reviews themselves are also subject to under-reporting and misclassification. The study validated the number of facility-based maternal deaths reported to the district level via comparison with a detailed accounting of facility health management information system (HMIS) and chart data and patient registers. It also validated the coverage and completeness of maternal death reviews in the study districts by 1) comparing facility records to objective evidence that a review was conducted, and 2) calculating the number of evidenced, complete maternal death reviews as a proportion of all facility-based maternal deaths reported. Finally, it tabulated maternal death review coverage using primary data for the numerator and denominator to the official value reported in the indicator in each country. Accounting for death review quality reduced the value of the indicator dramatically. Validation revealed substantial underreporting of the number of facility maternal deaths to the district, although the overall value of the indicator for reported maternal death review coverage did not change dramatically when calculated using maternal death data from district records as compared to facility death data due to congruence in underreporting both the numerator and denominator, reflecting inaccurate and incomplete reporting. Stratification by women’s sociodemographic factors suggested systematic differences in completeness of reviews by women’s age, place of residence, and timing of death. The study uncovered incompleteness in death reviews recorded at facilities and reported to districts from facilities; notably, very few met global quality standards for completeness. The value of the calculated indicator masked inaccuracies in counts of both deaths and reviews and gave no indication of completeness, thus undermining the ultimate utility of the measure in achieving an accurate measure of coverage. Ensuring that every maternal death is counted, and furthermore audited to learn how to prevent future maternal deaths, is critical to accelerating progress to ending preventable maternal mortality. Valid data on both the numbers of maternal deaths and review coverage will support countries in meeting the targets set forth in the SDGs.

The article by Gausman et al, “Revising the definition of “demand satisfied for family planning”: A cross-sectional study to explore incorporating person-centered constructs of demand, choice, and satisfaction” [20] 10.1371/journal.pone.0316725 explored whether the standard measure, which aggregates data from individual women but then uses a macroeconomic lens to look at contraceptive supply and demand, correlates well with women’s own subjective perceptions of their personal demand for contraception through modern methods or how well that demand has been satisfied. This potential discrepancy has been the subject of substantial debate, recently heightened due to increased attention to respectful care. Specifically, the inclusion of positive experiences of care is included as an indivisible element of quality in the current WHO definition of quality of care. Met need for family planning has important implications for maternal health and survival. A 2012 Lancet report using WHO maternal mortality estimates and data on contraceptive prevalence from the 2010 UN World Contraceptive Use database estimated that an additional 104,000 deaths per year could be averted by fulfilling unmet need for family planning (a 29% annual global reduction in preventable maternal deaths) [21]. This study aimed to assess whether women’s self-report of demand for family planning and satisfaction of demand converges with the standard DHS measure. Women’s autonomy in decision-making was assessed with the Family Planning Autonomy in Decision-Making (FP-ADM) scale. Adapted from the Mothers Autonomy in Decision-Making (MADM) scale [22], https://doi.org/10.1371/journal.pone.0171804, which measures autonomy in decision-making during maternity care, the FP-ADM scale measure autonomy in decision-making about contraceptive use and method within the context of contraceptive counselling. A psychometric validation of the Family Planning Autonomous Decision-Making (FP-ADM) scale using data from Argentina, Ghana, and India is presented [23]. https://doi.org/10.1371/journal.pone.0293586.

In Argentina and India, the percentage of women with demand satisfied after incorporating constructs of demand, choice, and satisfaction was substantially lower than that obtained using the standard definition. In Argentina, using the standard indicator, ~76% of sexually active women or married women had their demand for family planning satisfied, compared to 26.7% after incorporating demand, choice, and satisfaction. The change in the value of the indicator results both from adding constructs of choice and satisfaction, but also from including all women who indicated that they want to use a family planning method, regardless of marital status or sexual activity. In Ghana, there was little difference in value of the indicators using standard and alternative definitions. In all three countries, women categorized as having “demand satisfied” for family planning through the standard indicator were significantly less likely to use their preferred method of contraception, compared to those categorized as having demand satisfied through both standard and alternative indicator definitions. This study suggests the percentage of women with “demand satisfied” for family planning after incorporating person-centered constructs of demand, choice, and satisfaction is significantly lower than that obtained using the standard definition of this indicator. These results have implications for person-centered delivery of contraceptive counseling and services to improve dimensions of choice and satisfaction and may more accurately reflect women’s personal demand for contraception. Incorporating individual perspectives into a widely collected measure for tracking population progress may enhance quality of care in addition to contraceptive prevalence. Tracking demand satisfied for modern family planning supports countries in measuring performance of their family planning programs from a rights-based perspective. Examining whether current approaches to measuring demand satisfied reflect women’s perceptions of demand and satisfaction is important to furthering commitment to the principles of equity, informed choice, and voluntarism in family planning programs globally.

A final article by Gausman et al., “Comparative analysis of two approaches to measure SDG 5.6.2 in 75 countries: an indicator to monitor countries’ progress towards full and equal access to sexual and reproductive health care, information, and education” [24] 10.1371/journal.pone.0296382 was undertaken to address potential threats to the validity of the SDG indicator 5.6.2. related to its scoring methodology. This paper proposes a revised calculation of the indicator, exploring the values obtained with both scoring approaches and discussing the implications of systematic differences found for the interpretability of this important indicator for supporting countries and global efforts to achieve full and equal access to sexual and reproductive health and rights.

## Discussion

The collection of indicator validation research reports emanating from the IMHM Project contributes to ongoing efforts to strengthen measurement of upstream health determinants overall, and specifically in the context of maternal health. This work supports and aligns with a variety of current efforts aimed at improving maternal health measurement to better reflect the fundamental tenets enumerated in the Kirkland Principles and other calls to action [6, 7, 10, 11, 25–29]. Specifically, by identifying ways to improve the validity of “core indicators” to improve focus, ensuring their responsiveness to the needs of local, national and international users to improve relevance, addressing data quality and data sources to better reflect the needs of local populations and health systems, explicitly tracking equity, and fostering innovation, this body of work fully reflects the Kirkland principles.

**Fig 2:**
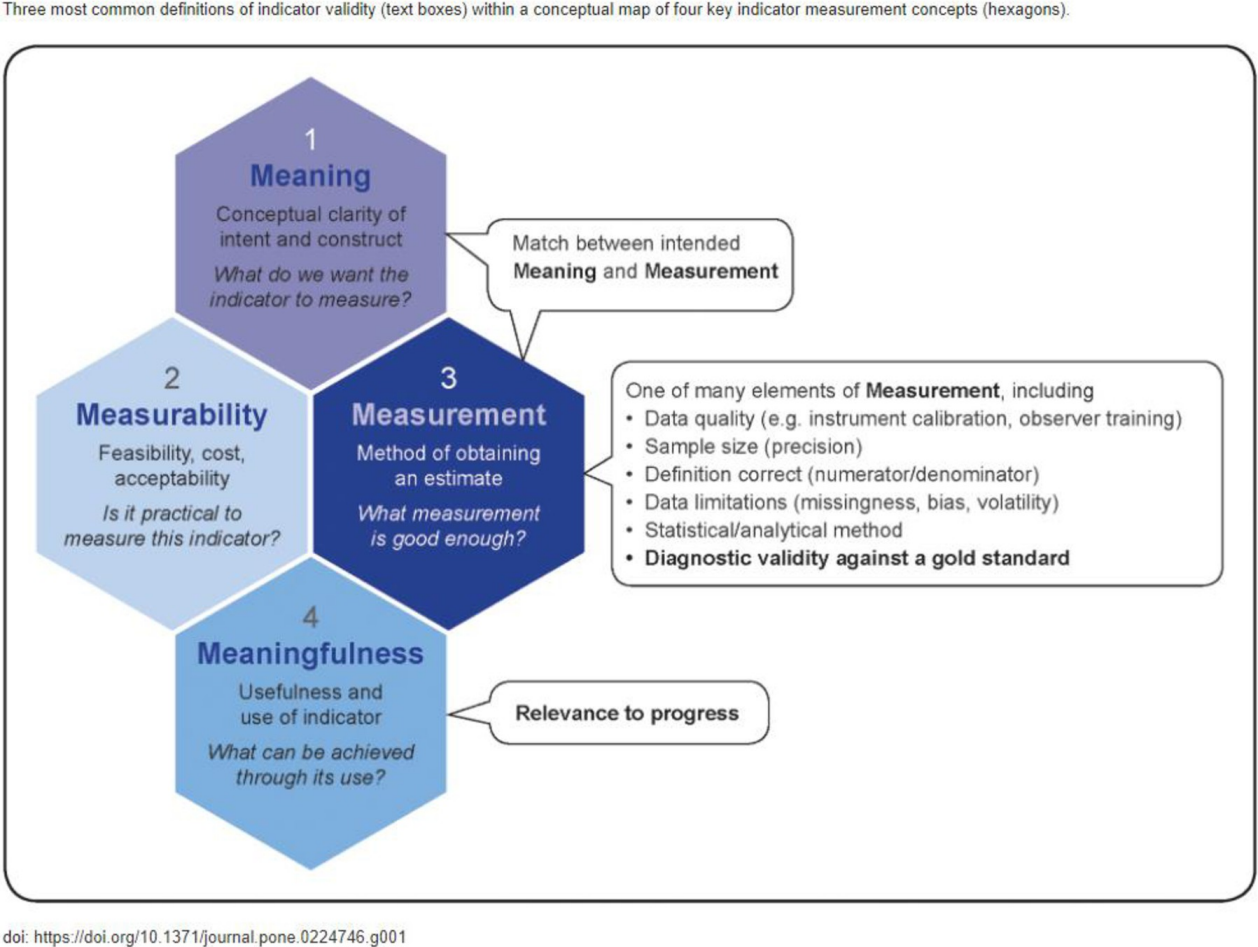
Diagram depicting common definitions of indicator validity mapped to key measurement concepts.

The findings of the multisite indicator validation research studies described and discussed in this collection make unique contributions to the field of maternal health, and to health measurement more broadly. From a measurement standpoint, this body of work adds to the knowledge base on methodologies for validating upstream social, economic, policy and health system-level indicators, applying theory or replicating approaches outlined in Benova et al. [11, 12] (**Fig 2**) [11] and will enable more reliable monitoring of distal determinants of maternal health. Dissemination of these findings to national and global measurement experts and decision makers will make information on indicator validity widely available for the first time, to assess and support national-level strategies and programs.

From a maternal health perspective, the IMHM Project research findings provide valuable evidence on health policy and health system performance, as well as actionable information about the underlying phenomena across the maternal health ecosystem that these measures seek to track, covering legal and regulatory frameworks, financing and Universal Health Coverage, effective coverage of essential personnel, services, and death reviews. Where these results highlight gaps between policy and practice, the information can be used to help bridge them.

The papers included in this collection also explore unique dimensions of the underlying constructs the indicators under study seek to measure and how successfully they are captured in their reported values, such as the effect of provider-level practice variation on legal “guarantees”, the inclusion of indirect costs and informal charges in the measurement of “free” care, and women’s own voices and perceptions in the measurement of their “demand satisfied” for family planning, among others. Several overarching themes emerge that unify the learnings from this body of work.

### Meaning and conceptual clarity

Problems with construct validity, defined as “whether a given operationalization (through indicator definition and its measurement) accurately reflects the phenomenon it is intended to measure” [12] were common for the policy and health system indicators studied. The findings highlight the importance of scrutinizing and pressure-testing the underlying assumptions about the concepts each indicator is intended to measure, how well those concepts are captured by its definition, whether the construct is a proxy for a related concept or phenomenon and if so, whether it captures the focal concept well enough. If assumptions do not hold up, the match between intended meaning and the outcome of measurement may be poor.

In the compiled reports, research findings challenged the assumption that the legality of abortion functions as a valid proxy for access to abortion on grounds for which it is legal. Similarly, the research showed that women bore direct and indirect costs and paid formal and informal fees for essential maternal health services that were designated free by national law or policy, thus challenging the construct of “free care”. Likewise, the measure of midwives’ authorization to perform basic emergency signal functions did not display convergence with their skills to perform those functions or their actual practice of them, questioning the construct validity of this measure.

### Measurement accuracy and performance

Accuracy of country data reported for many of the global policy indicators assessed was low when verified against the gold standard (national source documents). Moreover, for health system indicators looking at population coverage, the “correct” definition (numerator/denominator) is often uncertain/unknown. However, as shown in these studies, the value of an indicator may change based on the definition (for example, free of charge vs free of cost) or the data source (policy data vs surveys of hospital administrators vs. surveys of women), providing different information or interpretations of the underlying phenomena.

In general, this work shows that the validity of the value of the numerator is threatened when a simple count is used to estimate its value, without any measure of quality or functionality. The studies in this collection illustrate this point by showing how the estimate for the number of EmONC facilities varies when functionality is factored in, as previous studies have shown [30–32], but also when quality and completeness is factored into maternal death reviews, or when a measure of competency is factored into the measure of midwifery professionals in a district. For example, in India, the maternal death review coverage was 82.8% when considering maternal deaths for which there was evidence in facility records; however, this estimate decreased to 41.4% after including only maternal death reviews that met the definitional standard for quality and completeness. Similarly, in considering availability of EmONC, all provinces in Argentina reached or exceeded the target of one comprehensive EmONC (CEmONC) facility per 500,000 total population or 20,000 births when including all facilities designated as such in the numerator; however, only one province (Buenos Aires) reached the target when the numerator was restricted to facilities that provided obstetric and neonatal care 24 hours per day and 7 days per week with evidence that they had performed all CEmONC signal functions in the last 90 days.

Likewise, the optimal parameter for the denominator in measures of population coverage is often unknown and thus, the most readily available is used (e.g., total population). However, these studies show that these definitions matter greatly, because varying the numerator and/or denominator used can significantly alter the value of the indicator. For example, in one district in Ghana, the value of the indicator estimating density of midwives increased by 729% when comparing births instead of total population, while in a district in India, the value of the indicator increased by >8700%.

### Meaningfulness and utility/usefulness

Arguably, the ultimate assessment of an indicator’s validity is what can be achieved through its use. An indicator’s relevance for driving progress is based on the specific objective underpinning the decision to develop and track it, whereas its ability to actually drive toward these desired goals is predicated on the indicator’s validity. The indicators that were assessed for validity through this collection of studies were systematically prioritized and thoughtfully chosen as the strongest available measures to drive progress toward achieving the priority recommendations in the EPMM Key Themes [4] (Fig 1). Whether they are effective at doing so rests in large part on whether they can demonstrably fulfill the multiple dimensions of validity as it is understood in the field of maternal health measurement (Fig 2).

## Conclusions and recommendations

The overarching goal of the IMHM Project was to advance a robust, research-validated, field-tested monitoring framework for the EPMM Strategies, with the goal of supporting global and country-level efforts to improve maternal health. The project included research and technical work to strengthen the indicators focused on the distal determinants of maternal health and survival that emanated from the EPMM indicator development process (2016–2017) in contribution to achieving EPMM Key Theme 5.: Improve metrics, measurement systems, and data quality [4]. Efforts undertaken through the project included technical work to address challenges identified with components of specific indicator metadata, multi-country research to test and validate selected EPMM measures, and measure development to propose a number of additional measures to fill identified gaps. The project also supported seven multi-stakeholder national dialogues in which local stakeholders deliberated to self-identify priorities among the EPMM Key Themes for advancing toward ending preventable maternal deaths, and measures that could be taken to address them [33]. Improving global measurement of the social disparities, structural inequalities, and system deficiencies that continue to hamper progress in ending preventable maternal mortality is necessary to drive improvement and track progress towards achieving the priority recommendations outlined in the EPMM Strategies.

In sum, this body of work reveals important information about the performance of commonly used health system and policy indicators and the phenomena for measurement that they seek to capture. These upstream policy and health system-level determinants of maternal health are critical to address, but the available measures to track progress in these areas demonstrate significant threats to validity. Several key recommendations emerge for consideration and implementation by leaders of the maternal health measurement community at global and national levels:

- Adjust indicator definitions or add complementary measures as needed to capture the intended construct both accurately and fully.
- Pay careful attention to the choice of parameters for the calculation of indicator numerators and denominators, especially for population coverage measures, and factor in quality to estimate effective coverage.
- Examine the effect of data sources on the values achieved and consider those most appropriate for routine collection or for regular triangulation of results.
- Consider whether indicators with threats to validity can still drive improvement toward the measurement objective without masking lack of progress or giving unwarranted confidence that the goal is being achieved; establish when an imperfect indicator is good enough and when it is potentially harmful.
- Apply the Kirkland Principles for MNH measurement, with special attention to utility as defined by the end users.
- Look at global and national indicators as part of a more comprehensive strategy that includes alternative monitoring approaches (such as human rights reporting, social accountability programs, citizen monitoring, etc.) for tracking progress toward social and structural determinants that are hard to assess.
- Develop and evaluate innovative indicators to reduce important measurement gaps, such as assessment of service and provider distribution using Geographic Information Systems (GIS), understanding maternal health financing, and identifying priority areas for investment and measures to improve maternal health registration.
